# Neighborhood Cohesion and the Mental Health of Multimorbid Older Adults: CLSA Path Analysis Through Loneliness

**DOI:** 10.1093/geroni/igab046.945

**Published:** 2021-12-17

**Authors:** Daniel R Y Gan, Andrew Wister, John Best

**Affiliations:** Simon Fraser University, Vancouver, British Columbia, Canada

## Abstract

More older adults with multimorbidity are aging in place than ever before. Their mental health may be affected by housing and neighborhood factors. In this paper, we use structural equation modelling (SEM) to examine how the physical environment influences life satisfaction and depressive symptoms in two separate models. We included social environment (i.e., social support, social participation, walking) and loneliness as intermediate variables. Data were drawn from baseline and the first follow-up (after 3-4 years) of the Canadian Longitudinal Study on Aging (CLSA). Participants were N=14,301 adults aged □65 with □2 chronic illnesses. Good model fit were found after controlling for age, sex, education and baseline values (TFI=1.00; CFI=1.00; RMSEA<0.001; SRMR<0.001). The total effects of housing quality (Btotal=0.08,-0.07) and neighborhood cohesion (Btotal=0.03,-0.06) were weak but statistically significant in the expected direction. Together, the intermediate variables explained 21-31% of the total effects of housing quality and 67-100% of the total effects of neighborhood cohesion. Loneliness explains 27-29% of the total effects of physical environment on mental health, whereas walking explained a mere 0.4-0.9% of their total effects. Walking did not mediate between housing quality and mental health outcomes. Overall, the results support our path analysis framework: physical environment -> social environment -> loneliness -> mental health. Our model provided excellent explanations of the effects of neighborhood cohesion, especially on life satisfaction. If these associations reflect causal effects, community-based age-friendly interventions should focus on neighborhood cohesion and loneliness to promote the well-being of older adults who are aging in place with multimorbidity.

